# A green protocol for the one-pot synthesis of 3,4-disubstituted isoxazole-5(4H)-ones using modified β-cyclodextrin as a catalyst

**DOI:** 10.1038/s41598-022-23814-5

**Published:** 2022-11-09

**Authors:** Mahdieh Tajbakhsh, Mohammad Reza Naimi-Jamal, Saeed Balalaie, Mohadeseh Rezaeian

**Affiliations:** 1 Research Laboratory of Green Organic Synthesis & Polymers, Department of Chemistry, Iran University of Science and Technology, P.O. Box 16846–13114, Tehran, Iran; 2 Peptide Chemistry Research Institute, K.N Toosi University of Technology, P. O. Box 15875-4416, Tehran, Iran

**Keywords:** Catalysis, Organic chemistry

## Abstract

This manuscript reports an impressive and facile strategy for synthesizing isoxazole derivatives using immobilized Cu (I) in metformin-functionalized β-cyclodextrin as a catalyst. The architecture of this catalyst was characterized by different analytical techniques such as Fourier transform infrared spectroscopy, Thermogravimetric analysis, X-ray diffraction, Field emission scanning electron microscopy, and Energy-dispersive X-ray spectroscopy. The catalyst showed remarkable reusability even after 7 consecutive runs.

## Introduction

**Figure 1:**
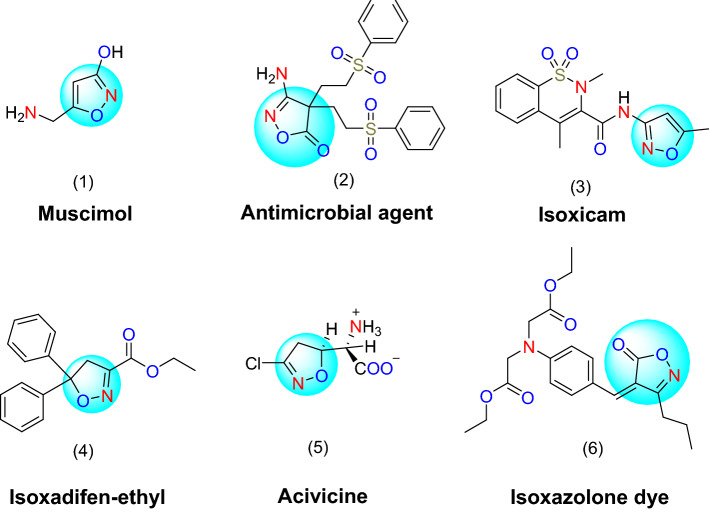
Examples of some isoxazole-containing bioactive compounds and drugs.

Isoxazoles contain a pyridine-like N-atom but differ from oxazoles by the presence of an N–O bond are important heterocyclic compounds, and possess various pharmacological activities^1^. A few isoxazoles exist in nature; Muscimol (1 in Fig. 1) is one of the main psychoactive constituents of *Amanita muscaria* and related species of mushrooms. Muscimol is a potent and selective orthosteric agonist for the GABA_A_ receptors. Among the synthetic isoxazoles, many biologically active compounds are found^2^. Some are important as drugs or biocides, such as the long-acting antimicrobial agent (2 in Fig. 1) and anti-inflammatory isoxicam (3 in Fig. 1). They are also used in agriculture as herbicides^3^, plant growth regulators, and fungicides^4,5^. A new class of substituted phenyl isoxazole derivatives was designed by an intermediate derivatization method as herbicide safeners (4 in Fig. 1)^6^, which is used to treat tuberculosis, and acivicine (5 in Fig. 1), an α-amino acid with antitumor activity. Different polymorphic forms of an isoxazolone dye were used as a filter dye in photographic films, and the polymorphic phase diagram was studied by researchers (6 in Fig. 1)^7^.

Notably, 4-arylmethylneisoxazol-5(4H)-ones are very useful synthetic intermediates of various applied heterocycles such as pyridopyrimidines^8^, 4-arylpyrrolidones, 1,3-oxazine-6-ones^9^, nicotinates^10^, β-alkylated γ-functionalized ketones^11^ and, α-aminopyrrole derivatives^12^. Therefore, these structures have interested organic chemists, and a review of the literature shows a wide variety of homogenous and heterogeneous catalysts and techniques such as sodium acetate^13^, DABCO^14^, modified-MMT^15^, Cu/TCH-pr@SBA-15^16^, L-valine^17^, ZSM-5^18^, DES (ChCl/Gly)^19^, ultrasonic irradiation^20^, Sn(II)-MMT^21^, and ionic liquids^22^. These include the cyclization of O-propioloyl oxime via intermolecular arylidene group transfer^23^, the condensation of 3-phenylisoxazol-5-one within aryl halide, the reaction of 1,3-dicarbonyl compounds with benzaldoximes, and condensation of hydroxylamine with β-keto esters. It was observed that the most common method for the synthesis of isoxazole involves one-pot three-component reactions of ethyl acetoacetate, hydroxylamine hydrochlorides, and aryl aldehydes using various catalysts, as mentioned above.

Cyclodextrins are natural substances that have relatively good solubility in hot water. However, β-CD is poorly soluble in cold water; thus, chemical modifications of β-CD are necessary to improve selectivity and solubility. These compounds stabilize linkers and metals with hydrophobic inner cavities and hydrophilic outer surfaces. Other attractive features include high availability, easy synthesis, large-scale production, and harmlessness. For these reasons, in recent years, cyclodextrins (CDs) and their derivatives have attracted much attention and have wide applications in various fields of science and technology^24^, including biosensors^25,26^, pharmacy, food industry, decomposition chemistry^27^, agriculture, and possible environmental protection^28,29^.

The catalytic behaviour of the functionalized β-CDs was studied in different organic reactions^30,31^ in different types, such as core–shell with Fe_3_O_4_^32^ or linking to other polymers and organic compounds. Pd@Aminopropanol- functionalized β-CD was used to catalyse the Suzuki reaction in 2018^33^. A green catalyst by functionalizing β-CD onto glass micro-particle surfaces was prepared in 2016 for selective oxidation of toluene to benzaldehyde^34^. β-cyclodextrin(β-CD) supported, hydroxyapatite encapsulated γ-Fe_2_O_3_ (γ-Fe_2_O_3_@HAp@β-CD) was successfully prepared and evaluated for the nucleophilic ring opening of epoxides in water for the preparation of β-azido alcohols, β-nitro alcohols, and β-cyanohydrins^35^.

Because of the importance of the isoxazole heterocycles, we explored an efficient, simple, and rapid synthesis of isoxazolones using a new functionalized β-CD as a homogeneous nanocatalyst. Water-soluble catalysts have been widely developed as efficient catalysts for organic reactions using greener methods because of environmental and economic considerations. Experiences from previous research on the production of triazole compounds^36,37^, as well as Ullman's reaction^38^ in our research group, have shown that the use of functionalized β-CD can be considered a new and attractive case study.

## Materials and methods

### Reagents and instrumentation

The reagents and solvents for the performed reactions like β‐cyclodextrin (98%) and metformin hydrochloride are commercially available and purchased from usual sources (Sigma-Aldrich and Merck), and were used without further purification. Copper (I) iodide salt was freshly prepared. All the reactions were monitored by TLC on pre-coated silica gel plates (0.25 mm) and visualized by fluorescence quenching at 254 nm. The melting points of the prepared derivatives were measured by an Electrothermal 9100 apparatus, which was reported without any correction. Elemental analysis was provided by EDX analysis, which was recorded by TESCAN4992. The FT-IR spectra were recorded in the range of 400–4000 cm^−1^ using the AVATAR spectrometer from Thermo company by using KBr pellets. The morphology of the synthesized nanocomposite was studied by SEM using MIRA2 TESCAN instrument. The TGA of the prepared nanocomposite was obtained by an STD Q600. The XRD measurements were recorded with the Rigaku Ultima IV.

### Catalyst preparation

**Scheme 1:**
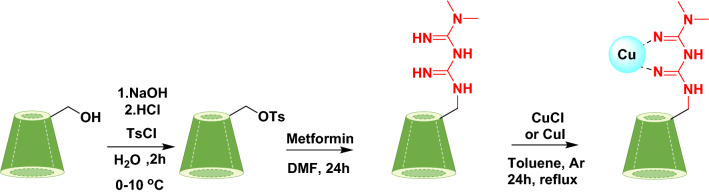
Catalyst preparation.

The catalyst was prepared according to the Scheme 1.

#### Synthesis of mono-6-(p-tosylsulfonyl)-6-deoxy-β-cyclodextrin (6-OTs-β-CD)^39,40^

β-Cyclodextrin (10.0 g, 8.8 mmol) was mixed with 100 mL deionized water at 0–5 °C, and 2–3 mL NaOH (8 M) was added dropwise over 5 min until the solution was completely clear. An amount of 0.2 g (1.1 mmol) *p*-toluenesulfonyl chloride dissolved in 10 mL of acetonitrile was added dropwise over 10 min, forming a white precipitate. After stirring for 2 h at room temperature, the precipitate was acidified to about pH 6–7 with HCl (6 M) and kept in a refrigerator at 0–4 °C overnight. The resulting white precipitate was obtained by filtration. The solid white product was recrystallized from hot water. Finally, the product was dried for 16 h at room temperature (Yield: 55%). IR: ν (cm ^−1^), 3367 (OH), 1641 (Ph-SO_2−_).

#### Synthesis of mono-6-(N-(N,N-dimethylcarbamimidoyl)-λ^*2*^-azanecarboximidamide)-6-deoxy-β-cyclodextrin (6-Met-β-CD)^41,42^

At this step, 1 g of 6-OTs-β-CD with 0.07 g metformin hydrochloride was dissolved in 4 mL of DMF, and a few drops (0.1 mL) of Et_3_N as a base were added to the above flask. The reaction mixture was stirred for 24 h in the reflux condition (a cream-yellow turbid solution was formed). Then, by adding 5–10 mL of acetone, a white precipitate was appeared. The precipitate was filtered through a Buchner funnel under vacuum, washed with fresh acetone twice, and stored for the next step^34^ (Yield: 35%).

#### Modification of Met-β‐CD with copper (I) chloride and copper (I) iodide (Cu@Met-β‐CD)

Various methods for making fresh copper (I) iodide salt have been reported^43,44^. By examining these methods, copper (I) iodide salt was freshly prepared with a slight change in the procedure in an easy, efficient, and cost-effective way^45^. Briefly, 0.5 g I_2_ (4 mmol) and 5 g NaI (33 mmol) were dissolved in 50 mL deionized water in a 100 mL round-bottom flask which was previously filled with a small amount of purified and polished Cu foil or granules. Then 2 drops of glacial acetic acid were added, and the reaction was carried out at 70–80 °C under vigorous stirring for 30 min. The change in the colour of the solution from brown to milky indicated a product's formation. The copper foil was removed entirely, and the reaction mixture was poured into a container of deionized water and ice and stirred for 10 min. Then, it was filtered and washed with plenty of water and acetone and dried in a vacuum oven. This product can be stored fresh for two weeks under argon gas. Finally, the obtained ligand 6-Met-β-CD was stirred with Cu (I) salt in dry toluene at reflux in an inert atmosphere (Ar or N_2_) for 24 h. The precipitate was filtered, washed with acetone, and dried at room temperature. In addition to copper (I) iodide, we also used copper (I) chloride salt to modify the Met-β-CD ligand. Comparisons of two modified catalysts showed that copper (I) iodide had better performance.

#### General procedure for the preparation of 3,4-disubstituted isoxazole-5(4H)-one

A mixture of ethyl acetoacetate (0.5 mmol), Cu@Met-β‐CD (0.03 g, 5 wt.%) as the catalyst, hydroxylamine hydrochloride (0.5 mmol), and aromatic aldehyde (0.5 mmol) was prepared and was stirred magnetically at 40 °C for 4–15 min (Table 3). The complete consumption of the starting materials was observed by TLC (n-hexane/ethyl acetate: 2: 1 v/v). After completing the process, the reaction mixture was extracted with EtOAc (3 × 10 mL). The organic phase was dried with anhydrous MgSO_4_, and the solvent was removed in vacuo to give the crude material. Most products did not require further purification and were only recrystallized in hot ethanol. The catalyst was dissolved in large amounts of water. To recycle the catalyst from the water, we added acetone to it, filtered the precipitate off, and dried it. The resulting product was obtained by filtration and washed with a cold ethanol–water mixture.

## Results

### Characterization of catalyst

#### FT-IR spectroscopy

**Figure 2:**
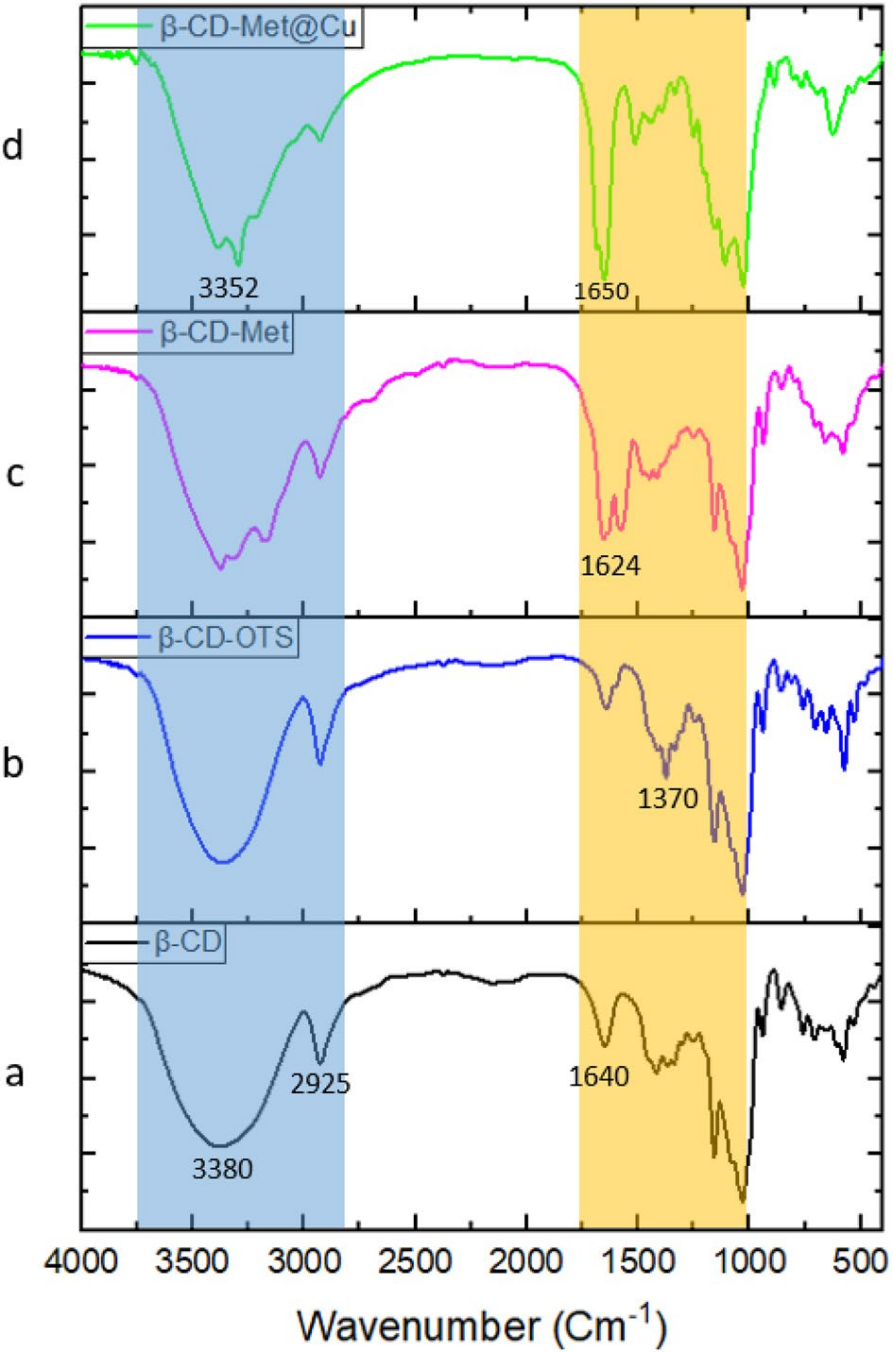
FT-IR spectra of (**a**) β-Cyclodextrin, (**b**) β-CD-OTs, (**c**) β-CD-Met, and (**d**) Cu@Met-β‐CD.

The Fourier transform infrared spectroscopy (FT-IR) was used to clarify whether β-CD is successfully covalently modified with metformin as a linker and, showed an interaction between Cu and linker. In Fig. 2a, the strong absorption bands at 3380 cm^−1^ and 1640 cm^−1^ correspond to OH groups' stretching and bending vibrations, respectively. The aliphatic CH absorption bands of cyclodextrin can be seen at 2925 cm^−1^. The peak in Fig. 2b at 1370 cm^−1^ corresponds to the characteristic bands of the S=O tosyl group. The peak of 1624 cm^−1^ in the Fig. 2c corresponds to stretching bonds C=N of metformin, which moved to 1650 cm^−1^ and changed the shape of the peak in Cu@Met-β‐CD upon complexation with copper (Fig. 2d). Also, N–H bonds stretching in 3500–3100 cm^−1^ and C-N bonds stretching in 1350–1100 cm^−1^ are observable (Fig. 2c). Further data approved the presence of metformin and copper as EDAX and ICP-OES.

#### EDAX and ICP analyses

**Figure 3:**
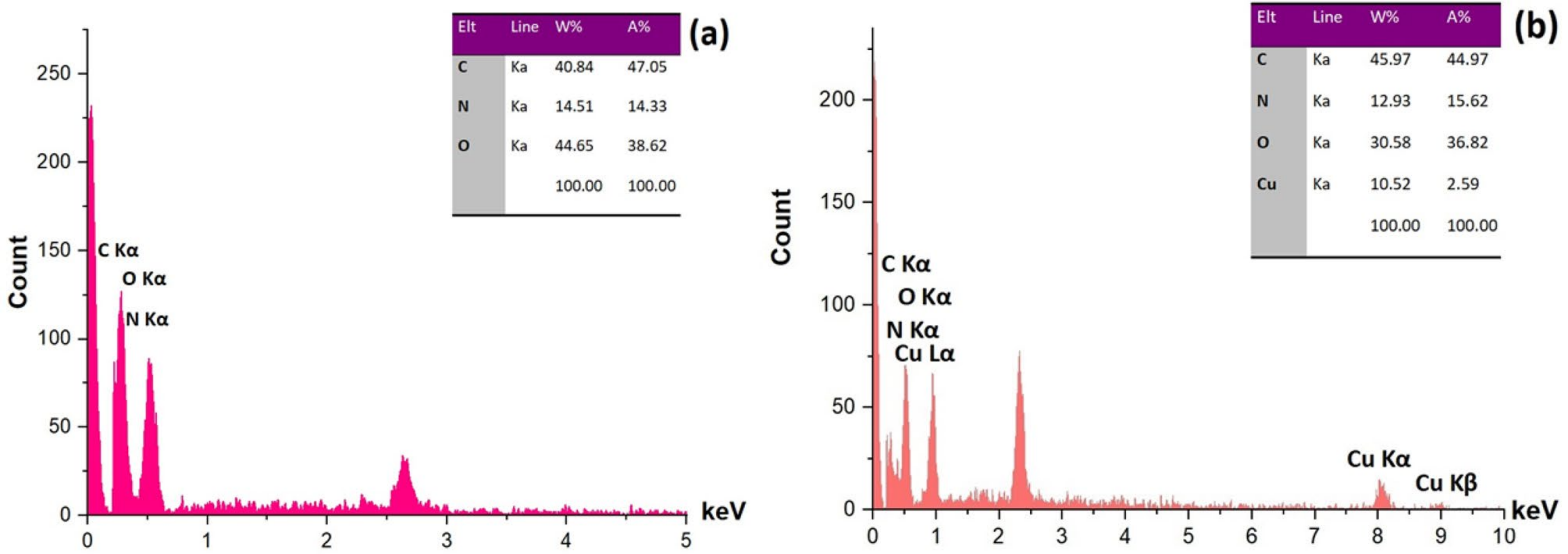
EDAX analysis of (**a**) β-CD-Met and (**b**) Cu@Met-β‐CD.

Energy Dispersive X-Ray Analysis (EDAX) was used to identify the elemental composition of (a) β-CD-met and (b) Cu@Met-β‐CD (Fig. 3). As expected, the nitrogen atom in the β-CD-Met and copper in the structure of the final catalyst is demonstrated. We confirmed the presence of the copper on the catalyst with the bands of 8.04, 8.90 keV (K lines), and 0.92 keV (L line). ICP analysis measured the exact amount of copper in the catalyst. This showed that the copper loading was about 0.07 mmol per gram of the Cu@Met-β‐CD.

#### Microscopic properties

**Figure 4:**
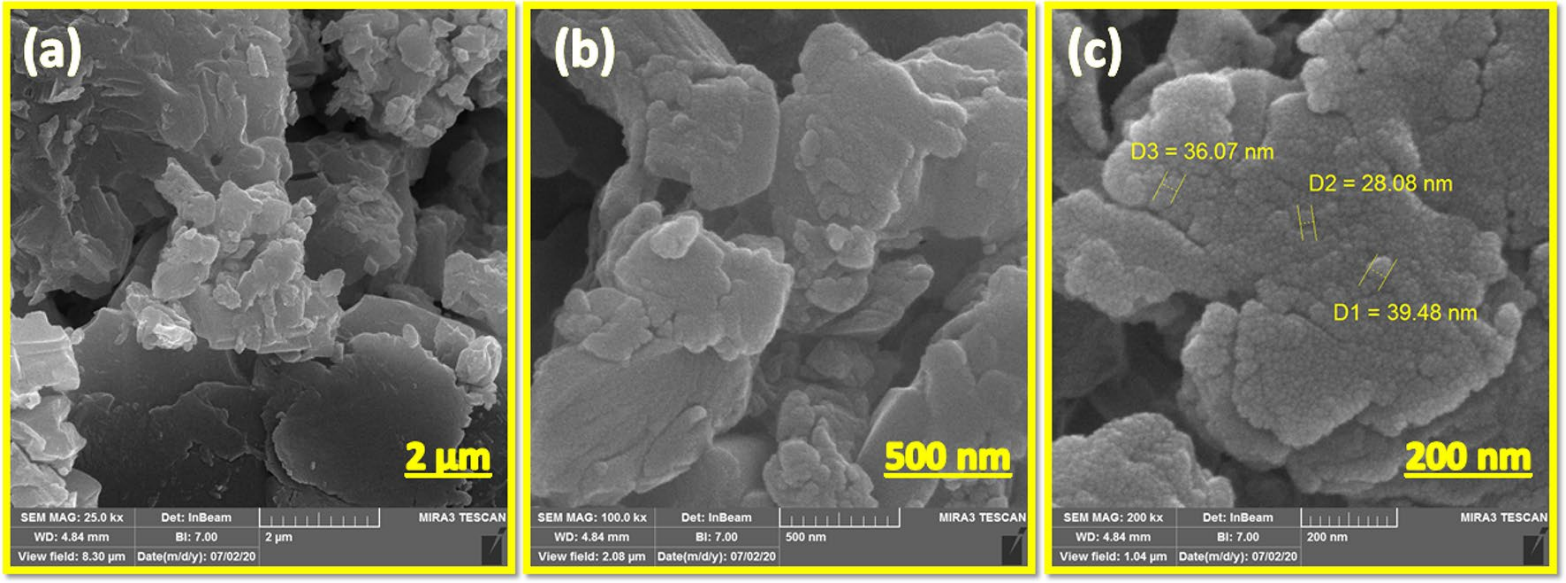
SEM images of Cu@Met-β‐CD.

Morphological studies of the inclusion complex Cu@Met-β‐CD and the size of the particles were also performed using scanning electron microscopy (SEM). The SEM images of the catalyst on 3 scales are shown in Fig. 4. It is observed that most parts of the sample exhibited spherical monodispersed round shape morphology. The diameter of the nanospheres is mostly in the range of < 50 nm.

#### TGA analysis

**Figure 5:**
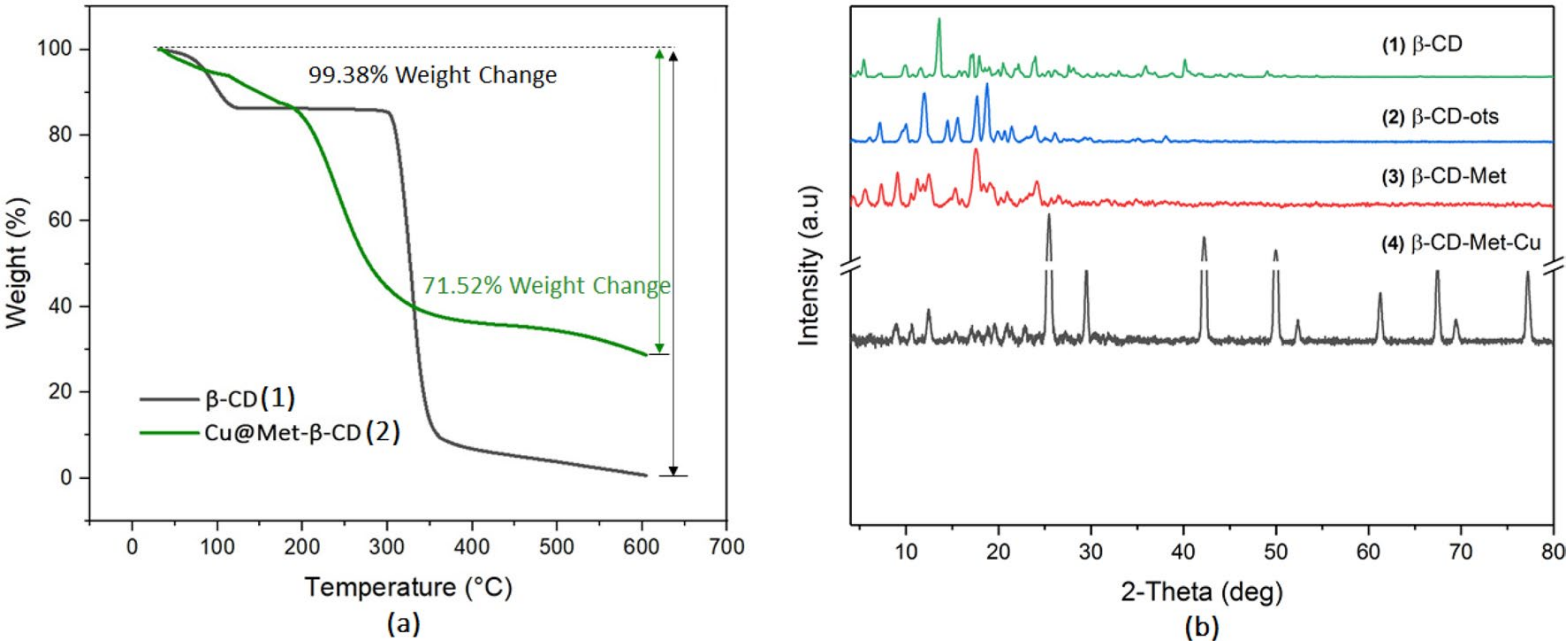
(**a**): TGA curves recorded in the air at a heating rate of 10 °C min^−1^, (**b**): XRD patterns.

The thermogravimetric analysis (TGA) was used to analyze synthetic materials' thermal stability and the content of active components. The TGA curves for (1) β-CD and (2) Cu@Met-β‐ CD are shown in Fig. 5a. Weight loss at temperatures less than 200 °C can be attributed to eliminating adsorbed water and other solvents. When heated to 600 °C, the weight loss can be attributed to the decomposition of the organic moiety. In the case of β-CD, the whole structure is decomposed up to 350 °C. In Cu@Met-β‐ CD, a weight loss of 71.52% occurred at temperatures of 200–350 °C.

#### XRD analysis

The XRD patterns of (1) β-Cyclodextrin, (2) β-CD-OTs, (3) β-CD-Met, and (4) Cu@Met-β‐ CD are presented in Fig. 5b. A scan efficiency of 0.1°S^−1^ was applied to record the powder patterns in the range of 3° ≤ 2θ ≤ 80°. These results are indicated by the XRD pattern of β-CD and its characteristic peaks with crystalline nature. Here is no noticeable change in the structure of β-Cyclodextrin after functionalization with metformin. As a result, the XRD patterns proved that the catalyst had been synthesized without damaging the crystal structure. Furthermore, the peaks at 2θ = 25.4°, 29.5°, and 42.2° in Cu@Met-β‐ CD could be indexed to the (111) and (200) planes of Cu, which is very close to the values in JCPDS– International Center for Diffraction Data.

## Discussion

### Application of the catalyst in the synthesis of isoxazole-5(4H)-ones

**Scheme 2:**
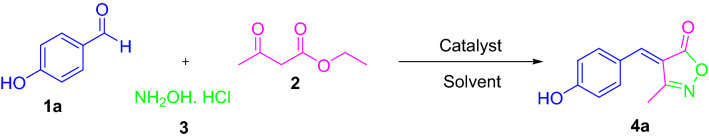
Synthesis of 3-methyl-4-Hydroxymethylene isoxazole-5(4H)-one as a model reaction.

A novel approach for the preparation of a stable and active Cu catalyst supported on functionalized β-CD has been reported in our research group, and its application was investigated for the synthesis of 3-methyl-4-arylmethylene isoxazole-5(4H)-ones via condensation of aldehyde derivatives with hydroxylamine hydrochloride and ethyl acetoacetate in mild conditions. As shown in scheme 2. At first, synthesis with 4-hydroxy benzaldehyde was selected as the model reaction, and according to Table 1. this reaction was investigated with different catalysts and conditions.

**Table 1:** Optimizing the catalyst amount and reaction condition.

Entry	Catalyst	Solvent	Time (h)	Temp. (°C)	Yield (%)
1	–	EtOH	8	r.t	Trace
2	Piperidine (20 mol%)	iPrOH	4	70	80
3	Boric acid (10 mol%)	EtOH	8	70	85
4	Et_3_N (20 mol%)	EtOH	4	70	75
5	Cu(II)@Met- β‐ CD (1%wt)	H_2_O	1	r.t	75
6	Cu(II)@Met- β‐ CD (2%wt)	H_2_O	0.5	r.t	80
7	Cu(II)@Met- β‐ CD (5%wt)	H_2_O	0.1	50	85
8	Cu(I)@Met-β‐ CD (1%wt)	H_2_O	0.03	r.t	75
9	Cu(I)@Met-β‐ CD (2%wt)	H_2_O	0.03	r.t	87
10	Cu(I)@Met-β‐ CD (5%wt)	H_2_O	0.06	r.t	90
11	Cu(I)@Met-β‐ CD (10%wt)	H_2_O	0.06	r.t	92
**12**	**Cu(I)@Met-β‐ CD (2%wt)**	**H**_**2**_**O**	**0.06**	**50**	**97**
13	β-CD-Met	H_2_O	6	70	35

Significant values are in bold.

Experiments have shown that this reaction produces very little product in the absence of a catalyst for 8 h (Table 1, Entry 1). By adding basic catalysts such as piperidine (Table 1, Entry 2), triethylamine (Table 1, Entry 4), or boric acid as acidic catalysts (Table 1, Entry 3) and optimizing the conditions with higher temperature, the reaction proceeded at less time. To compare the performance of Cu(I) and Cu(II), some of the catalysts were prepared with Cu(OAc)_2_.5H_2_O and tested with different amounts in the model reaction (Table 1, Entry 5–7). The results show that the performance of the catalyst is better in the presence of Cu(I) as a Lewis acid in the synthesis reaction of isoxazoles, the yield of the products is higher, and also less time is required to carry out the reaction. Different amounts of Cu(I)@Met-β‐CD as a green catalyst were used to synthesize 3-methyl-4-Hydroxymethylene isoxazole-5(4H)-one and the results shown in the table were obtained. Experimental searches have shown that increasing the amount of catalyst by more than (10%wt = 130 mg) does not significantly affect the product yield (Table 1, Entry 8–12). Raising the temperature had a negligible effect on the reaction efficiency. Finally, 50 °C and 2% wt catalyst values were selected as the optimal reaction conditions (Table 1, Entry 12). Copper-free functionalized Met-β‐CD catalyst was also investigated in this reaction, and it was found that the role of copper as Lewis acid is significant (Table 1, Entry 13). Comparing the efficiency of the synthesized catalyst with previous reactions, we found the catalyst has the necessary and sufficient efficiency in performing this reaction.

Various solvents were tested for this reaction, but water was selected as the green solvent (Table 2). Due to the solubility of the catalyst in water and the homogeneity of the catalyst, the yield of the products was better.

**Table 2:** Solvent optimization.

Entry	Solvent	Temp. (°C)	Yield (%)
1	iPrOH	70	90
2	EtOH	50	85
**3**	**H**_**2**_**O**	**50**	**97**
4	CH_3_CN	50	80
5	Toluene	100	85
6	CH_2_Cl_2_	r.t	70
7	Solvent-free	20	10

Significant values are in bold.

Under the optimized conditions, a study on various aldehydes was carried out, and the representative results are presented in Table 3. According to this Table, a broad range of aromatic aldehydes, including electron-withdrawing (4b, 4 m) or electron-donating (4a, 4 g, 4e) substituent aldehydes, were transformed into the corresponding isoxazole-5(4H)-ones in excellent yields.

**Table 3:**
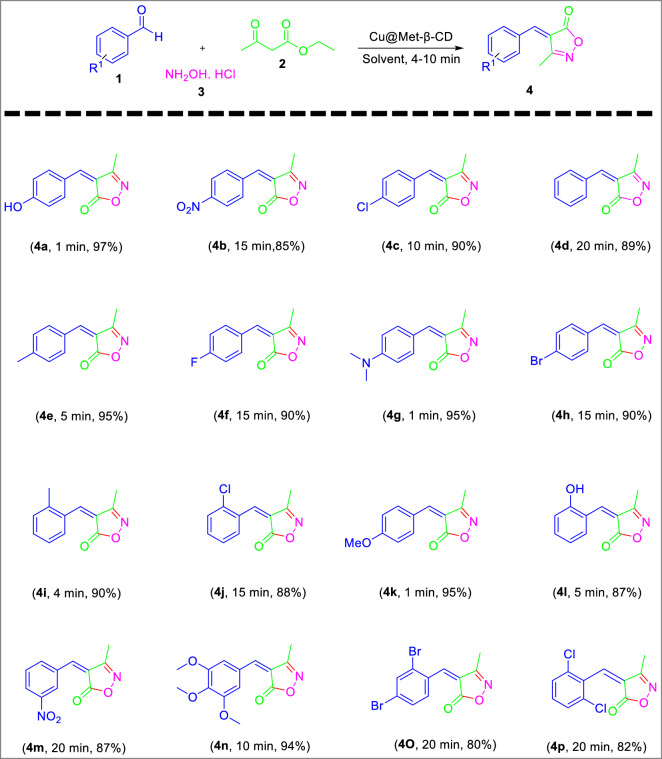
Preparation of 3-methyl-4-arylmethylene-isoxazole-5(4H)-ones in the presence of Cu@Met-β‐ CD as a green catalyst.

### Mechanisms of the reaction

**Scheme 3:**
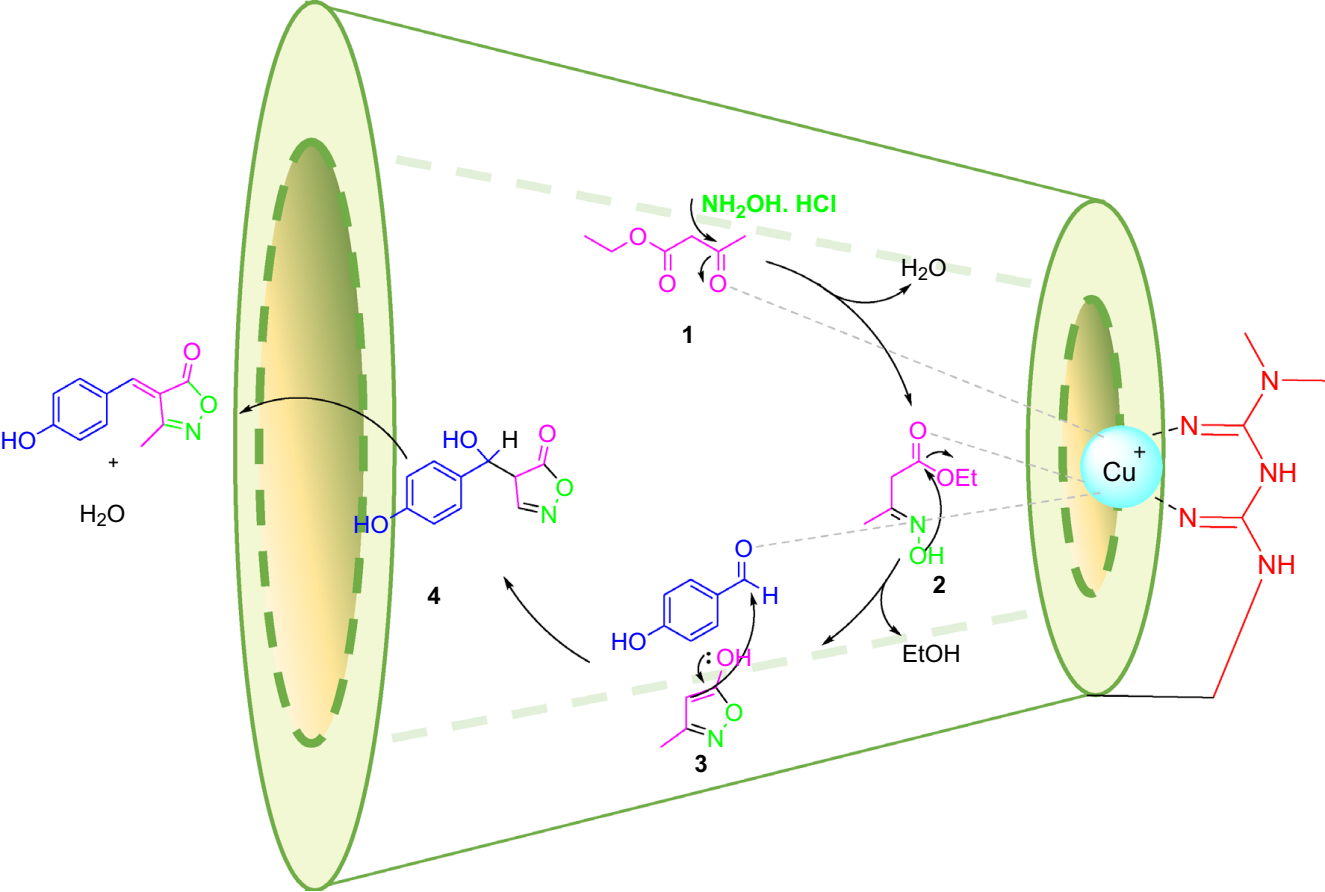
Proposed mechanism for the synthesis of 3-methyl-4-benzyl methylene-isoxazole-5(4H)-ones in the presence of β-CD-Met@Cu.

At first, the Cu immobilized in functionalized β-CD acts as a Lewis acid and increases the electrophilic character of the carbonyl groups in ethyl acetate (Scheme 3). Then the nucleophilic attack of the amino group of hydroxylamine hydrochloride occurs at the activated carbonyl carbon of ethyl acetoacetate to result in oxime intermediate **2**. The condensation gives Isoxazol-5-ones as the heterocyclic compound **3**. Isoxazol-5-ones are characterized by relatively high acidity at C-4 (pK_a_ 4 − 6). The resulting carbanions find wide use as nucleophiles, which can also be used in condensation reactions with aldehydes to generate electrophilic arylidene isoxazole-5-ones **4**. The effect of the catalyst on the carbonyl group of an aldehyde increased the electrophilic property.

### Comparison with other catalysts

To show the capability and efficiency of this method and the Cu@Met-β‐ CD as a suitable catalyst, a comparison has been summarized in Table 4 with the previous methods for synthesis of 3-methyl-4-arylmethylene-isoxazole-5(4H)-ones. As indicated in Table 4, this method avoids the disadvantages of other procedures, such as excess reagents and long reaction times.

**Table 4:** Comparison of Cu@Met-β‐CD and various catalysts in the synthesis of 4-(4-hydroxybenzylidene)-3-methylisoxazol-5(4H)-one.

Entry	Catalyst	Condition	Time (min)	Yield (%)	Ref
1	Sodium acetate	EtOH/H_2_O, r.t	30	90	^13^
2	DABCO	EtOH, r.t	30–180	87	^14^
3	Modified-MMT	H_2_O, 70 °C	7–70	92	^15,46^
4	Cu/TCH-pr@SBA-15	Solvent-free, 80 °C	8	95	^16,47^
5	L-Valine	EtOH, reflux	1–240	95	^17^
6	ZSM-5	Solvent-free, 100 °C	15–30	96	^18^
7	DES (ChCl/Gly)	H_2_O, 60 °C	20	95	^19^
8	Na_2_S	EtOH, r.t	150	80	^20^
9	Sn(II)-MMT	H_2_O, 30 °C, U.S	10–50	96	^21,46^
10	Hydroxyapatite	H_2_O/EtOH, r.t	20	97	^48^
11	Ionic liquid	Solvent-free, 70 °C	30–50	94	^22^
12	Nano-MgO	H_2_O, r.t	80–120	90	^49^
13	Sulfated polyborate	Solvent-free, 80 °C	15–60	90	^50^
14	PPTS	H_2_O, reflux	60	80	^47,51^
15	**Cu@Met-β‐CD**	**H**_**2**_**O, r.t. up to 50** °C	**2–5**	**97**	**This work**

Significant values are in bold.

### Recyclability of catalyst

**Figure 6:**
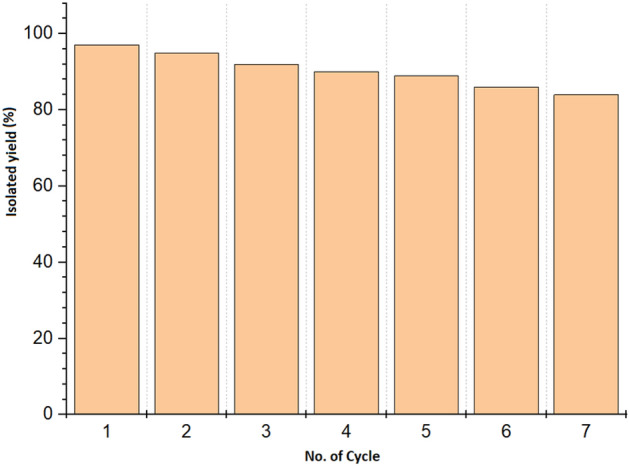
Recycling of the catalyst, in the synthesis of 4-(4-hydroxybenzylidene)-3-methylisoxazol-5(4H)-one.

The recovery and ability to reuse of the Cu@Met-β‐CD as a green catalyst was tested several times (Fig. 6) in the synthesis of 4-(4-hydroxybenzylidene)-3-methylisoxazol-5(4H)-one as a model product. After each run, the product was extracted from an aqueous solution with ethyl acetate. After adding acetone, the catalyst was easily recovered by precipitating from the solution, and filtered out after the reaction. The filtrates were dried in a vacuum and the resulting catalyst was reused directly for the next run. The ICP-OES analysis of the filtrate did not detect a significant amount of the leaching of copper species at the 3rd stage of the recyclability study of the catalyst (≤ 3 ppm). The results indicated that the recovered catalyst was still enough active without a significant loss of its performance. At the end of the seventh cycle, a yield of 88% of the product has been achieved. The decrease in the product yield may be due to the fact that the catalyst is partially lost during reuse.

**Figure 7:**
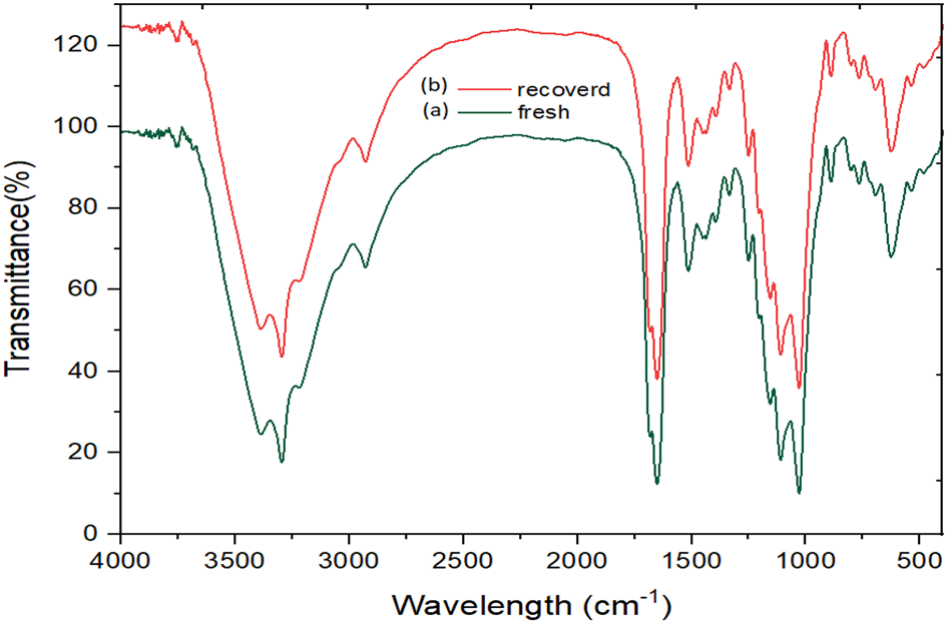
FT-IR spectrum of the nano-catalyst (**a**) and recovered Cu@Met-β‐CD (**b**).

The recovered nanocatalyst structure was confirmed with FT-IR spectroscopy. Figure 7 shows that there is no difference in the FT-IR spectra of fresh and the seven-times reused catalysts.

## Conclusion

In summary, we describe here a new, efficient protocol for the synthesis of 3-methyl-4-arylmethyleneisoxazol-5(4H)-ones by a three-component reaction between aromatic aldehydes, ethyl acetoacetate, and hydroxylamine hydrochloride catalyzed by Cu@Met-β‐CD as a benign catalyst. It is a commercially available, inexpensive, supramolecular, biodegradable, and reusable catalyst. The essential advantages of this method are simplicity of the procedure and, clean work up without column chromatography, good to excellent yields, short reaction times, and the use of non-toxic green solvent. It is an environmentally friendly process.

## Supplementary Information

Supplementary Information.

## Data Availability

All data generated or analysed during this study are included in this published article and its supplementary information files.
