# Supplementary material for: A green protocol for the one-pot synthesis of 3,4-disubstituted isoxazole-5(4H)-ones using modified β-cyclodextrin as a catalyst
Source: Sci Rep. 2022 Nov 9;12:19106. doi: 10.1038/s41598-022-23814-5 (PMC9646907; doi:10.1038/s41598-022-23814-5)
Supplement: Supplementary file 1 — Supplementary Information. [file 41598_2022_23814_MOESM1_ESM.docx]

**Supplementary information**

**A green protocol for the one-pot synthesis of 3,4-disubstituted isoxazole-5(4H)-ones using modified β-Cyclodextrin as a catalyst**

*Mahdieh Tajbakhsh ^a^, Mohammad Reza Naimi-Jamal ^a, *^, Saeed Balalaie ^b^, Mohadeseh Rezaeian ^a^*

*a. Research Laboratory of Green Organic Synthesis & Polymers, Department of Chemistry, Iran University of Science and Technology, P.O. Box 16846–13114 Tehran, Iran*

*b. Peptide Chemistry Research Institute, K. N. Toosi University of Technology, P. O. Box 15875-4416, Tehran, Iran*

**Corresponding author E-mail:* [*naimi@iust.ac.ir*](mailto:naimi@iust.ac.ir)

**Table of Content**

FT-IR spectra of some products (**4a, 4e, 4k, 4l**) …………………………………………….……(S2-S5)

^1^H-NMR spectra of some products (**4a, 4k**) …………………………………………………………(S6-S7)

FT-IR spectrum of the reused catalyst…………………………………………………………………S8


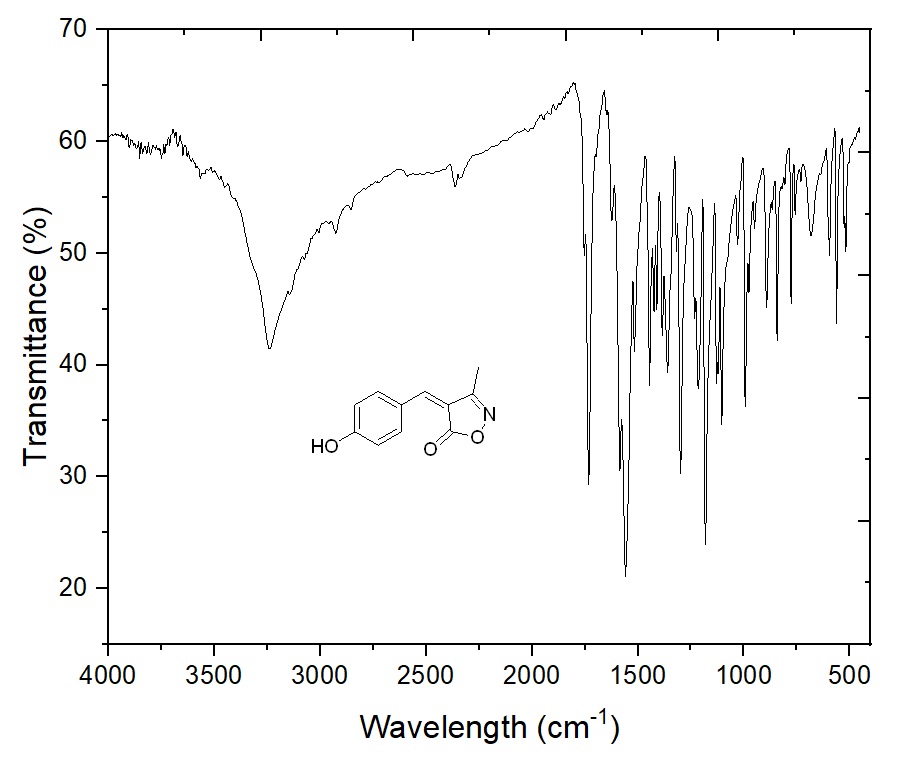


Figure S1: FT-IR spectrum of product 4a


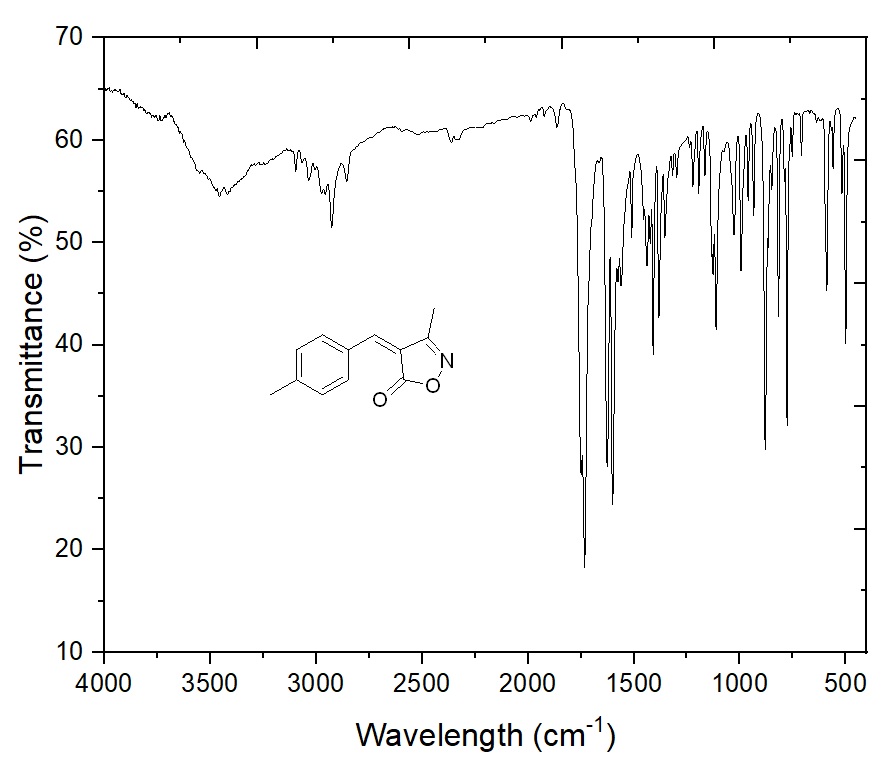


Figure S2: FT-IR spectrum of product 4e


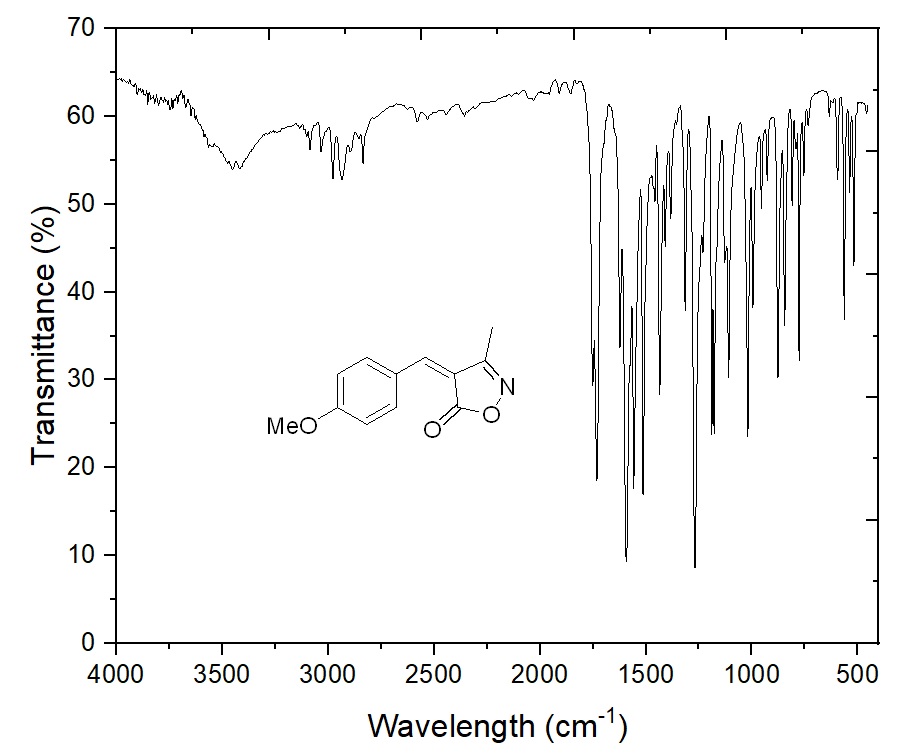


Figure S3: FT-IR spectrum of product 4k


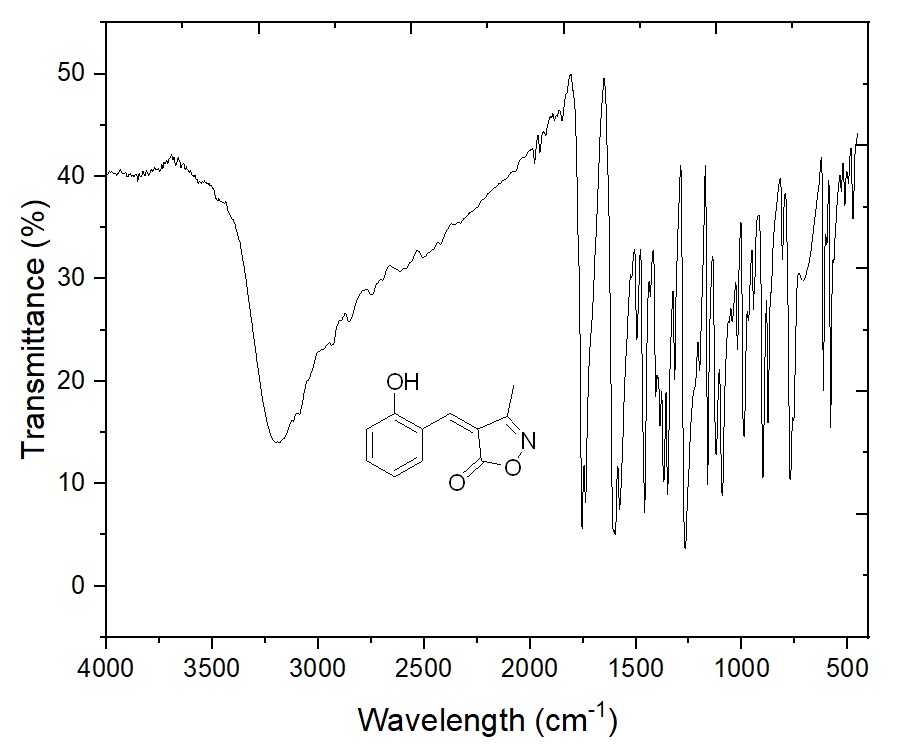


Figure S4: FT-IR spectrum of product 4l


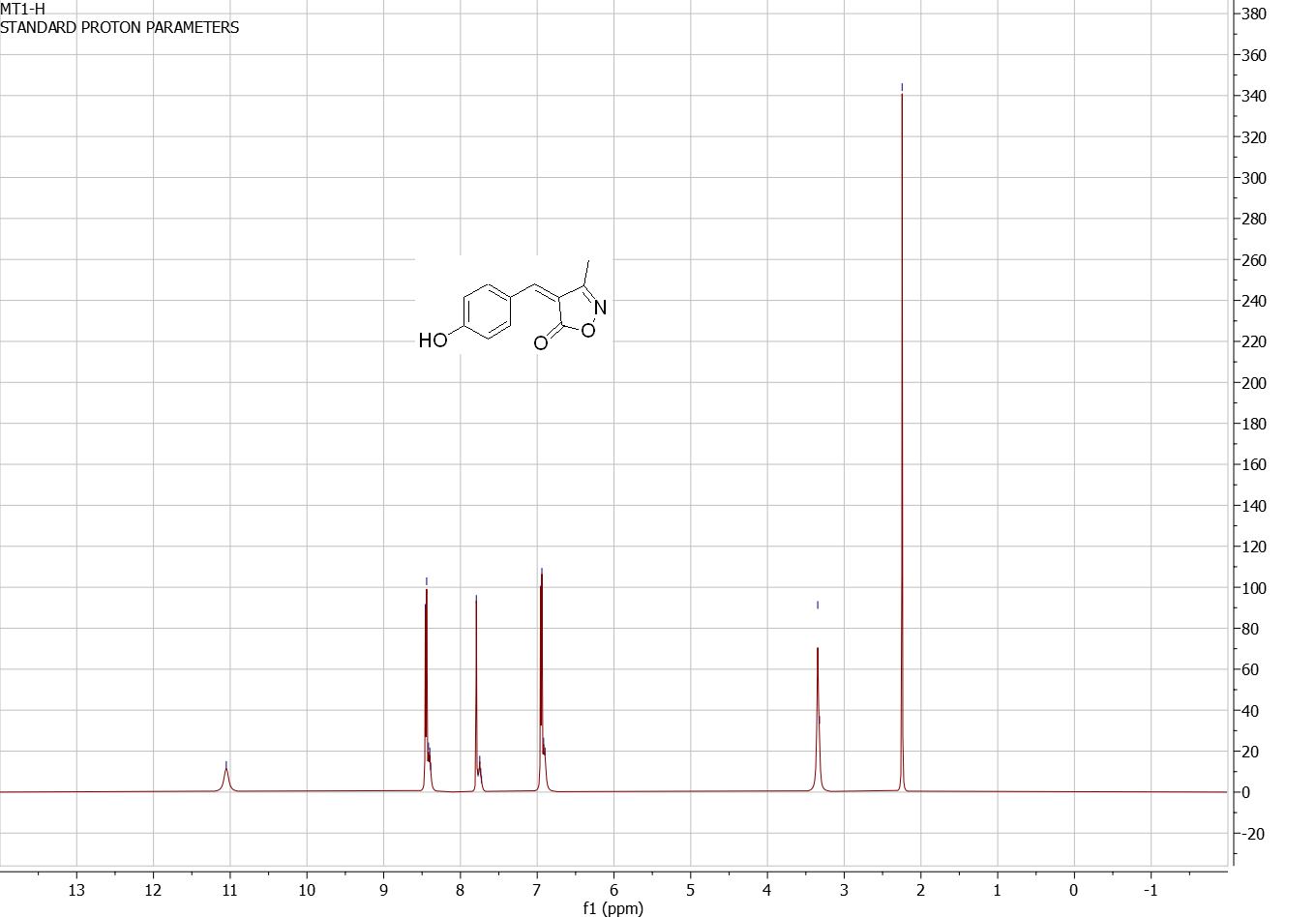


Figure S5: ^1^H-NMR spectrum of product 4a


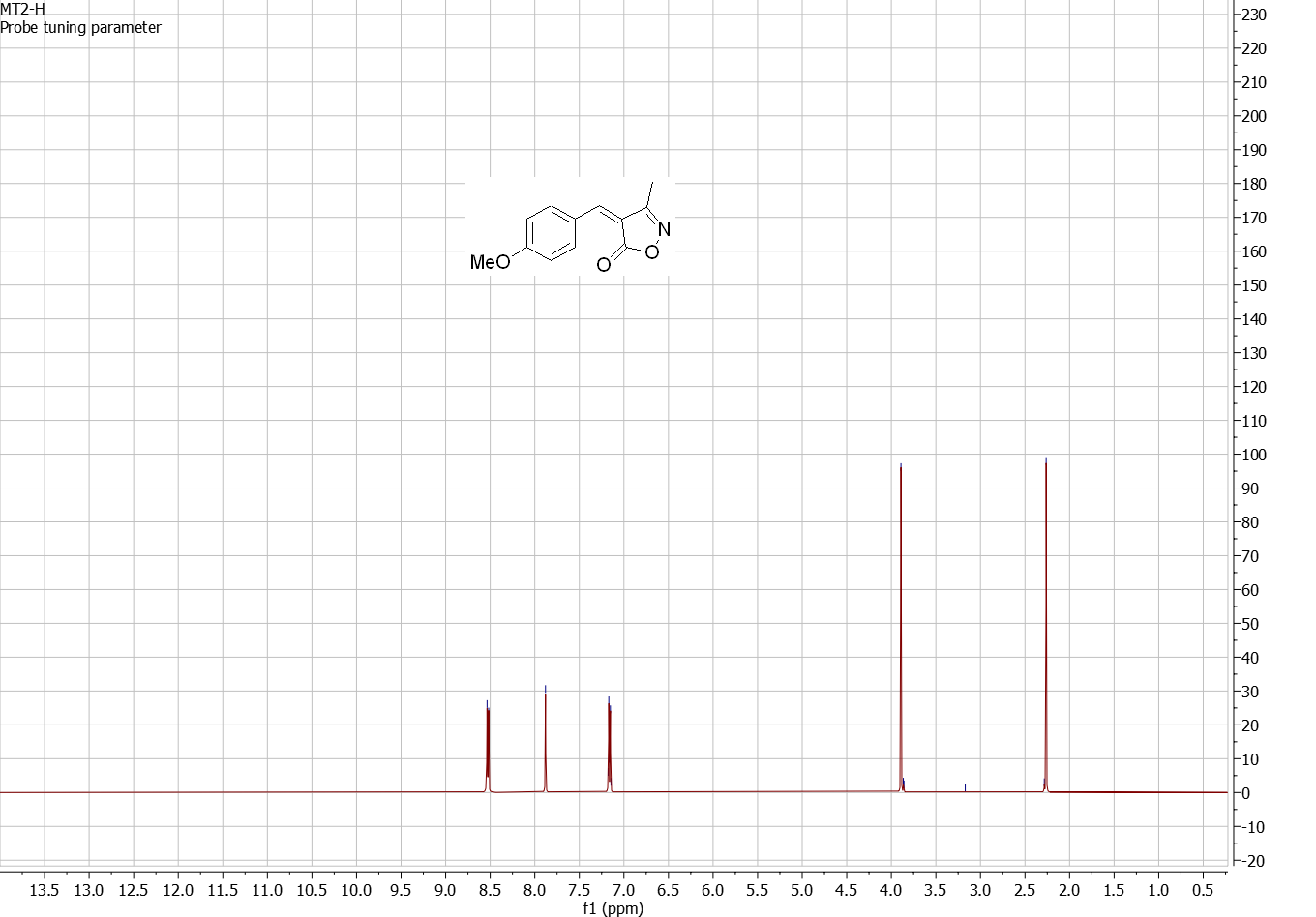


Figure S6: ^1^H-NMR spectrum of product 4k


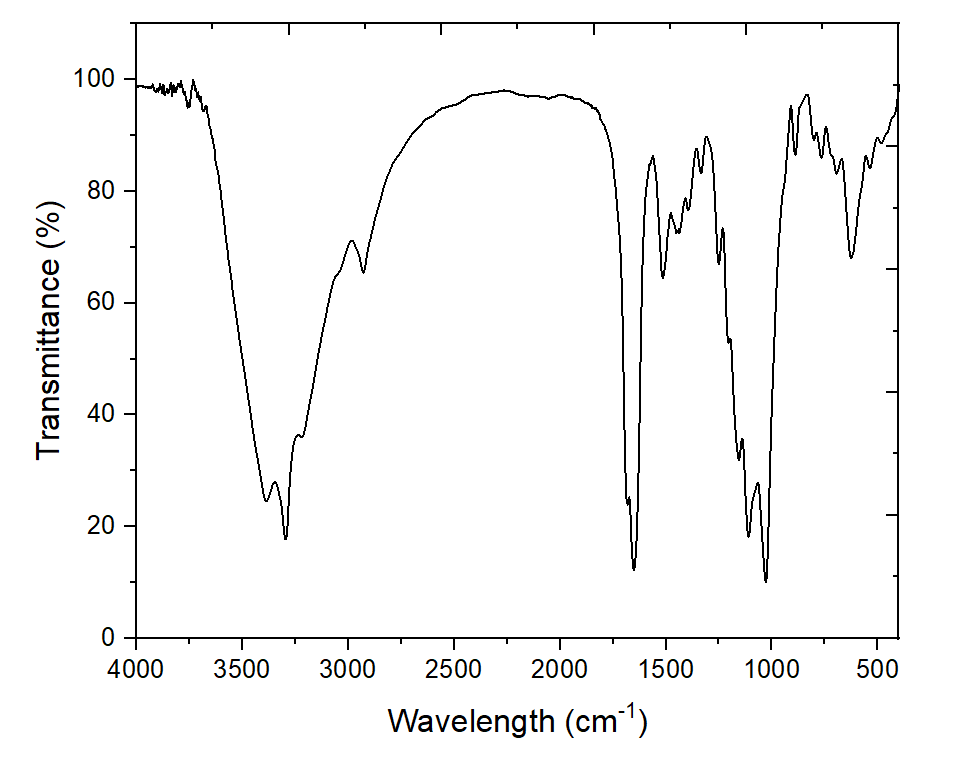


Figure S7: FT-IR spectrum of the reused catalyst
